# Antioxidant Capacity, Lipid Oxidation, and Quality Traits of Slow- and Fast-Growing Meagre (*Argyrosomus regius*) Fillets During Cold Storage

**DOI:** 10.3390/antiox14020124

**Published:** 2025-01-21

**Authors:** Ioannis Mittakos, Cosmas I. Nathanailides, Lambros E. Kokokiris, Alexandra Barbouti, Konstantina Bitchava, Evangelia Gouva, Markos N. Kolygas, Michael A. Terzidis, Michael G. Kontominas

**Affiliations:** 1 Department of Agriculture, Faculty of Agriculture, University of Ioannina, 47100 Arta, Greece; i.mittakos@uoi.gr (I.M.); egouva@uoi.gr (E.G.); kolygasmarkos@gmail.com (M.N.K.); 2 Laboratory of Chemical Biology, Department of Nutritional Sciences and Dietetics, International Hellenic University, 57400 Sindos, Thessaloniki, Greece; lamprosk@ihu.gr (L.E.K.); mterzidis@ihu.gr (M.A.T.); 3 Department of Anatomy-Histology-Embryology, Faculty of Medicine, University of Ioannina, 45110 Ioannina, Greece; abarbout@uoi.gr; 4 Laboratory of Applied Hydrobiology, Department of Animal Science, School of Animal Biosciences, Agricultural University of Athens, 11855 Athens, Greece; bitchava@aua.gr; 5 Department of Chemistry, Faculty of Natural Sciences, University of Ioannina, 45110 Ioannina, Greece; mkontomi@uoi.gr

**Keywords:** intrinsic antioxidant activity, polyunsaturated fatty acids, lipid oxidation

## Abstract

Meagre (*Argyrosomus regius*) is an important species in aquaculture, with size and flesh quality playing key roles in its production and marketability. This study aimed to examine the relationship between growth and flesh quality parameters, including fatty acid content (FA), total antioxidant capacity (TOAC), superoxide dismutase (SOD), lipid oxidation (LO), muscle cellularity (MC), and filleting yield (FY) during cold storage. Fish from the same hatchery raised under identical conditions showed size variation after 12 months. Fish below 600 g were classified as slow growing (SG), while those above 1000 g were classified as fast-growing (FG). The results showed that FG fish had higher body weight, moisture, and FY but exhibited lower levels of fat and polyunsaturated fatty acids (PUFA). SG fish had higher TOAC and SOD activity, which significantly declined during cold storage in both groups but remained higher in SG fish. Despite the higher lipid content in SG fish, no significant differences in malondialdehyde (MDA) levels, an indicator of LO, were observed between the two groups, suggesting that the elevated antioxidant defenses in SG fish mitigated lipid peroxidation. This study underscores intrinsic antioxidants’ potential to preserve lipid quality of fish fillets during cold storage.

## 1. Introduction

Consumers are increasingly discerning in their seafood choices, prioritizing options that offer both product sensory and nutritional value. The global demand for fish species rich in high quality protein and ω-3 fatty acids has surged, reflecting a growing health consciousness among consumers [1]. Meagre (*Argyrosomus regius*) has gained considerable attention in the aquaculture sector due to its favorable farming attributes, including rapid growth and adaptability as well as its desirable sensory attributes. These attributes, closely related to consumer acceptance, make meagre a sought-after species. Renowned for its high protein content, low fat levels, and essential nutrients, such as ω-3 polyunsaturated fatty acids (PUFAs), meagre appeals to health-conscious consumers [2,3]. At the same time, the impressive aquaculture performance of meagre, marked by rapid growth rates, short production cycles, and broad market acceptance, solidifies its position in Mediterranean aquaculture [4,5].

Τhe market size of aquacultured meagre typically starts at approximately 500 g. However, larger sizes are also frequently available, particularly those exceeding 1 kg, all of which are obtained after 12 months’ time, catering to the growing demand for premium-quality fish in diverse culinary markets. The availability of a broad weight range contributes to the versatility and economic viability of meagre aquaculture, enabling it to meet market demands across different sectors from local fresh fish markets to high-end restaurants [1,2,3]. A notable challenge within several aquaculture fish species is the significant variability in individual growth rates, hindering production efficiency maximization. Even within the same fish farm, growth rates can vary significantly due to genetic and environmental factors as well as resource competition. Such a variability affects the composition of the fish’s muscle tissue, raising questions for producers and consumers alike regarding potential flesh quality differences between fish of different sizes [6,7].

From a fish farm management perspective, growth variability leads to differences in harvested fish size and lipid quality, necessitating labor-intensive size grading of farmed fish. After size grading, fish from a common pond or floating cage must be sorted into different ponds or floating cages according to their size, resulting in increased demand for resources to ensure uniform product quality and marketability [6,7,8]. In terms of consumers’ perspective, several studies suggest that fish proximate composition may vary with size and age [9,10]. Growth rate variability may also affect fillet proximate composition, with faster-growing fish potentially accumulating more lipids or showing differences in muscle fiber size and density [11], important parameters for texture and water holding capacity of fish flesh. Understanding these differences is crucial for various aquaculture aspects, including selective breeding, feed formulation, and product quality management, to enhance production efficiency, product consistency, and sustainability. Furthermore, variations in lipid quality and quantity among individual fish, i.e., fast growing vs. slow growing, may affect post-harvest lipid oxidation, off-flavor development, and fillet shelf life [12,13,14,15]. This, in turn, impacts the nutritional value and overall product quality.

Lipid oxidation significantly impacts the quality and shelf life of fish fillets, especially those rich in omega-3 polyunsaturated fatty acids, which are highly susceptible to oxidative degradation. Lipid oxidation alongside muscle cellularity—defined as the size and number of muscle fibers (myofibers) within a myotome—play a pivotal role in determining the oxidative stability of fish fillets. Larger myofibers, for instance, have a greater capacity to retain water, directly impacting product drip loss and the extent of oxidative changes post-harvest [15]. Unlike mammals, fish muscle lacks complex connective tissue and relies on segmented blocks (myotomes) for structure. This structural simplicity increases susceptibility to postmortem stiffening (rigor mortis), drip loss, and oxidation, ultimately compromising product texture and oxidative stability [16,17]. The interplay between lipid and protein oxidation further accelerates quality degradation, as reactive lipid oxidation products, such as aldehydes, can react with proteins, amplifying oxidative damage in muscle tissue [18], while the interplay between oxidation processes and other key parameters, such as proximate composition, muscle growth, and muscle cellularity, further highlights the complexity of fish flesh quality. Growth rate is inherently linked to lipid content and fatty acid composition, as faster growth often correlates with higher intramuscular fat deposition, which may increase susceptibility to oxidation if not managed appropriately [16]. Similarly, proximate composition—including protein, fat, moisture, and ash content—not only provides insights into the nutritional value of the fish but also influences the oxidative stability of the fillets [13,19]. Higher fat content, particularly polyunsaturated fatty acids (PUFAs), enhances the nutritional value of the fish but also increases the risk of lipid oxidation, necessitating strategies to improve oxidative stability [1,12] and lipid quality of fish fillets [1,19,20,21]. Lipid oxidation of muscle cells represents a key challenge in the preservation of biological membranes, where it can propagate oxidative reactions and compromise structural integrity. These alterations weaken lipid–lipid and lipid–protein interactions, compromising membrane integrity of muscle cells. In the absence of antioxidants, lipid oxidation can accelerate, contributing to post-harvest quality losses in fish fillets. Intrinsic antioxidants present in fish fillets, collectively measured as total antioxidant capacity (TOAC), play a critical role in delaying lipid oxidation. TOAC encompasses both enzymatic antioxidants, such as superoxide dismutase (SOD), catalase, and glutathione peroxidase (GPx), and non-enzymatic antioxidants, including vitamins C and E. These antioxidants act synergistically to neutralize reactive oxygen species (ROS) and interrupt the chain reactions that propagate oxidative damage. Nevertheless, the protective effects of TOAC diminish over time, antioxidant molecules are gradually depleted, while the activity of several enzymes, including antioxidant enzymes, declines during prolonged storage [22,23,24].

Although rapid growth is often considered a desirable trait in aquaculture, the nutritional quality of the resulting fillets, particularly regarding lipid composition and omega-3 fatty acid content, remains underexplored. In some aquaculture fish species, growth and lipid quality are associated; it can be hypothesized that growth rate significantly influences lipid quality, omega-3 fatty acid composition, and oxidative stability in meagre fillets, with potential implications for their shelf life during cold storage. For example, it is reasonable to hypothesize that differences in growth rate may result in differences in lipid content and muscle cellularity with consequences for lipid oxidation during cold storage [11,16]. Therefore, differences in growth rate may not only affect lipid content and muscle structure but also increase the risk of lipid oxidation, making the role of intrinsic antioxidants critical, as they can be expected to at least partially mitigate lipid oxidation initially.

The aim of this study was to investigate the potential relationship between growth rate and muscle composition in farmed meagre, focusing on fatty acid content, intrinsic antioxidant capacity, oxidative stability, as well as other important fish quality parameters during cold storage in relation to prevention of oxidative damage, particularly within the context of lipid quality preservation, ensuring both stability and enhanced shelf life in aquaculture products.

## 2. Materials and Methods

### 2.1. Fish Samples and Somatometric Traits

Fish were obtained in June 2022 from a commercial fish farm in northwestern Greece, originating from the same batch and grown under identical conditions in floating cages. All fish in this study were 12 months of age, ensuring uniformity in terms of age. They were fed a commercial diet (48% protein, 16% fat, 3% fiber, 8.3% ash). Despite weekly monitoring and adjusted feeding rates, high variability in body weight after 12 months highlighted the need for refined growth management. To address this, size grading was implemented, categorizing fish into ”small” and “large”, and fish were subsequently placed in different floating cages. For the needs of our research, subsamples of small and large fish were further categorized as slow growing (SG) and fast growing (FG); fish weighing 450–650 g were classified as SG, while 1000–1200 g were defined as the FG category. This approach ensured the “small” group included fish in the lower half of the size distribution, and the “large” group captured those above the median.

**Figure 1:**
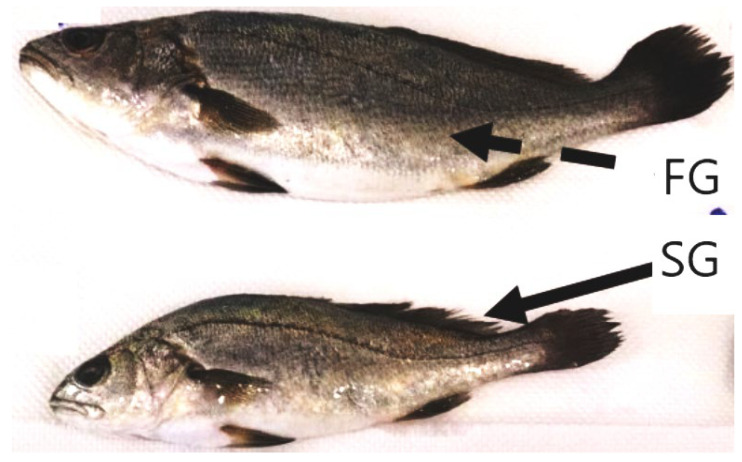
Representative samples of slow-growing (SG) and fast-growing (FG) meagre used in the present study.

Samples from two cages per growth group were selected, and random subsamples (*n* = 20) were analyzed for body length, weight, testis, viscera, and liver weight, providing a comparative assessment of slow-growing (SG) and fast-growing (FG) fish. A visual comparison of the slow-growing (SG) and fast-growing (FG) fish can be seen in Figure 1**.**

The sampling was conducted during the summer, a period that typically coincides with the peak of gonadal development in older fish, when reproductive organs are more pronounced and easier to differentiate between males and females. In fact, sexual maturation in *Argyrosomus regius* generally occurs at weights well above 1 kg between the second and third year of life, with studies indicating that 50% of males reach maturity at approximately 2.7 years of age [25].

However, due to the young age of the specimens in this study (1+ year age group), the gonads were barely visible. Macroscopic examination classified them as stage I of gonadal development; this stage is characterized by thread-shaped testes that range in color from white to rose or brown, appearing either translucent or opaque. These testes have a small but discernible width and thickness and are far smaller than sexually mature fish [23,24]. To avoid any sex-related growth differences [25,26,27], only male fish were used; all specimens were screened for the presence of male gonads, as identifying female gonads at this stage proved challenging. At this stage (immature), the lobes of the ovary are thin and dark red, while the gonadal lobes of the testes are thin with a creamy white appearance and relatively easy to identify by macroscopic examination.

Physiological and quality indices determined in SG and FG samples included gonadosomatic index (GSI) = gonad weight (g) × body weight ^−1^ (g) × 100; hepatosomatic index (HSI) = liver weight (g) × body weight^−1^ (g) × 100; viscerosomatic index (VSI) = visceral weight (g) × body weight^−1^ (g) × 100; and perivisceral fat (PVF) = perivisceral fat weight (g) × body weight^−1^ (g) × 100.

Samples of white epaxial muscle tissue were collected; the left side of the fillets was used for histological analysis, and the right side was used for proximate composition, fatty acid profile (FA), total antioxidant capacity (TAOC), oxidative stability (lipid oxidation), and muscle cellularity analysis.

### 2.2. Proximate Composition and Fatty Acid Content

For proximate composition analysis, the moisture, protein, lipid, and ash contents of deep white muscle tissue obtained from the upper epaxial anatomical region of the skeletal musculature were determined at the first day of sampling according to AOAC (2005) [28] using samples of 3 pools of 5 fish per group. Similarly, the analysis of FAs was performed once in 3 pools of 5 fish per growing group. Lipids were extracted using a 2:1 chloroform and ethanol solvent ratio and converted to their corresponding fatty acid methyl esters (FAMEs) directly from freeze-dried muscle tissue under acidic conditions. FAs were methyl-esterified using a 12% boron trifluoride in methanol solution (BF3-MeOH). The resulting methyl esters were extracted with hexane. The methyl ester analysis was performed by using a Chromatec-Crystal Model 9000 (Yoshkar-Ola, Russia) chromatograph equipped with a flame ionization detector and an Agilent HP-88 (88-cyanopropyl)-aryl-polysiloxane) column (100 m × 0.25 mm i.d., 0.2 μm film). A sample of 1 μL was injected with a 1:20 split ratio at 250 °C. Oven temperature was programmed to increase from 120 °C to 180 °C in 10 min (6 °C/min), then kept at 180 °C for 10 min, increased to 220 °C in 5 min (8 °C/min), kept at 220 °C for another 5 min, increased to 240 °C in 5 min (4 °C/min), and finally kept at 240 °C for 4 min. The detector was set at 280 °C. Helium was used as the carrier gas at a constant flow of 2 mL/min. The identification and quantification of the fatty acid methyl esters were performed based on the Supelco 37 fatty acids methyl ester standard purchased from Sigma Aldrich (CRM47885). Methyl ester of behenic acid was added as the internal standard.

### 2.3. Total Antioxidant Capacity, SOD Activity, and Lipid Oxidation

The process was conducted repeatedly over a 10-day period using cold-stored (2 °C) fish fillets. At each time point, muscle tissue samples were collected and analyzed to track changes in total antioxidant capacity (TAOC), superoxide dismutase (SOD) activity, and lipid oxidation. All assays were performed in duplicate at 20 °C.

Total antioxidant capacity (TAOC) was determined by preparing muscle tissue samples according to the manufacturer’s instructions for the Antioxidant Assay Kit (CS0790, Sigma-Aldrich, Germany), as modified by Fernando et al. [29] for fish tissues. Muscle tissue samples from three pools of three fish per group were diluted (1:10 *v*/*v*) in Tris-HCl buffer, homogenized, and centrifuged at 12,000× *g* for 5 min. The resulting supernatant was collected and stored at −80 °C until analysis. The TAOC was measured spectrophotometrically using a Beckman DU-50 series spectrophotometer at a wavelength of 405 nm. The assay is based on the oxidation of 2,2′-azino-bis(3-ethylbenzothiazoline-6-sulfonic acid) by a ferryl myoglobin radical, where the degree of oxidation is inversely proportional to the antioxidant activity. Trolox was used as a standard, and TAOC values were expressed as mM Trolox/mg.

For the SOD assays, white muscle tissue samples were excised from three pools of five fish each and immediately placed on ice. The tissue from each pool was minced and homogenized separately in ice-cold 50 mM phosphate buffer (pH 7.4) containing 1 mM EDTA. Superoxide dismutase (SOD, EC 1.15.1.1) activity was assayed as described by Antonopoulou et al. [30]. The assay was performed at 20 °C in a medium containing 800 μL of 50 mM phosphate buffer (pH 7.4), 55 μL of a 100/50 mM EDTA/Mn solution, 40 μL of 7.5 mM NADH solution, and 100 μL of 10 mM β-mercaptoethanol solution. The decrease in NADH absorbance at 340 nm was monitored, and the extinction coefficient for NADH (ε = 6.22 mM) was used to calculate the rate of NADH oxidation. One unit of SOD activity was defined as the amount of tissue extract required to reduce the NADH oxidation rate of the control by 50%, and the activity was expressed as units per milligram of protein.

Lipid oxidation was measured using a previously described protocol [15] based on malondialdehyde (MDA) and determined spectrophotometrically using the thiobarbituric acid (TBA) method (n = 3 pools of 5 fish each per group). The concentration of TBA reactive substances (TBARS) was expressed as mg MDAKg^−1^ of tissue wet weight.

### 2.4. Muscle Ceullularity

In order to evaluate muscle cellularity, samples from the white epaxial muscle of the post-anal caudal area were collected (5 fish per group) and processed for routine histological processing. The samples were fixed in 10% phosphate-buffered formaldehyde saline, subsequently dehydrated in a graded series of ethanol solutions, and embedded in paraffin. Transverse sections were obtained (6 μm thick) and stained with H&E stains. The cross-sectional area (CSA) of at least 150 individual myofibers from each fish was traced using a computerized image analysis system (Seescan plc, Cambridge, UK). The density of myofibers in each sample of a cross-sectional area was estimated by using the ratio of the cross-sectional area divided by the mean myofiber size [31].

### 2.5. Chemicals

Histological consumables (4% formaldyhye, histology grade paraffin wax, and xylene) were purchased from Biognost Ltd., Zagreb, Croatia. All other chemicals and reagents used were ACS grade and were purchased from Sigma-Aldrich: boric acid; boron trifluoride-methanol solution; copper (II) sulfate pentahydrate; hydrochloric acid; methanol; n-hexane; phosphotungstic acid; potassium hydroxide; potassium sulfate; sodium chloride; sodium hydroxide; sulfuric acid; TBA; and trichloroacetic acid.

### 2.6. Statistical Analysis

For the statistical analysis, the data were first tested for normality using the Shapiro–Wilk test. The mean values of the variables between groups were compared using a *t*-test (for body traits and muscle quality indices) or one-way ANOVA (for TOAC, SOD, MDA, and muscle size classes). When ANOVA revealed significant differences, post hoc comparisons were performed using Tukey’s honest significant difference (HSD) test to identify specific group differences. All statistical analyses were performed using IBM SPSS Statistics (v. 22). The significance level was set at α = 0.05.

## 3. Results and Discussion

### 3.1. Body Traits and Proximate Composition of Muscle Tissue

The analysis of body traits in slow-growing (SG) and fast-growing (FG) meagre revealed significant differences (Table 1). While both groups had similar viscerosomatic (VSI) and hepatosomatic (HSI) values, the FG fish displayed a significantly higher body weight (BW) and gonadosomatic (GSI) index value but lower perivisceral fat (PVF) (*p* < 0.05). Filleting yield (FY) was higher *(p* < 0.05) in FG fish (Table 1).

The lipid and moisture contents differed significantly between groups. FG fish had significantly higher moisture (80.40 vs. 78.29%, Table 1) and significantly lower lipid contents than SG (0.27 vs. 0.78%, *p* < 0.05, Table 1). Protein and ash contents were similar (*p* > 0.05) between groups, averaging around 19% and 1% (Table 1).

Analysis of body traits and proximate composition indicated that slow-growing and fast-growing meagre exhibited distinct growth and metabolic characteristics. FG fish showed lower fat deposition and higher reproductive investment (*p* < 0.05), as indicated by the GSI%. These differences could have implications on flesh quality. FG fish, for example, exhibited less perivisceral and muscle fat (*p* < 0.05) compared to SG fish. These factors may affect the product sensory attributes and consumer appeal. The present values of moisture and fillet yield are similar to those of Grigorakis et al. [30], who reported a filleting yield (38.03%) and moisture (80.40%) for reared meagre. With respect to fat content, the results of the present study are lower than those of Grigorakis et al. [30] (1.06%) and Giogios et al. [1] (1.09% and 0.73% for small and large reared meagre, respectively). Differences in fat content between the two studies may be attributed to the fact that, in the present study, fat determination was carried out on deep white muscle, which contains substantially less fat compared to red muscle tissue. Dorsal muscle samples typically contain a mixture of predominantly white muscle fibers but also some red muscle fibers, which are known to have higher fat content [31]. This mixture could introduce variability, making it more challenging to obtain consistent results. Focusing on deep white muscle ensures that the fat content analysis is more reliable, as this tissue is the most abundant and uniform in terms of composition.

In a similar study on the flesh quality of farmed meagre, Saavedra et al. [32] reported pronounced differences in fat content between farmed meagre of different weights. In smaller fish (800 g), the fat content was 1.3%, roughly half of that for 1500 g (2.5%) and 2500 g (2.9%) fish. In contrast, with regard to physiological indices, Martelli et al. [33] reported similar gonadosomatic (0.15), hepatosomatic (1.20), and viscerosomatic (4.78) index values for large meagre harvested in May but substantially higher fillet yields (50.64%) compared to those of the present study.

### 3.2. Fatty Acid (FA) Analysis

The analysis of FAs revealed significant differences between meagre growing groups. FG meagre had significantly higher contents in palmitic (*p* = 0.013), stearic (*p* = 0.016), palmitoleic (*p* = 0.000), dihomo-γ-linolenic (*p* = 0.002), and eicosatrienoic acids (*p* = 0.014) (Table 2). However, SG meagre had a similar content in eicosapentaenoic (EPA, *p* > 0.05) but a significantly higher content in docosahexaenoic acid (DHA) compared to FG meagre (*p* = 0.033). The most abundant fatty acids in both FG and SG were oleic, palmitic, docosahexaenoic, linoleic, and nervonic acids. A similar trend regarding the abundance of fatty acids was also recorded by Grigorakis et al. [30], Giogios et al. [1], and Saavedra et al. [32].

FG meagre had significantly lower contents in omega-3 (ω-3) FAs (15.52 vs. 24.30, *p* = 0.001, Table 3), PUFA (31.35 vs. 38.38, *p* = 0.016), and ω-3/ω-6 ratio value (1.07 vs. 1.78, *p* = 0.021). However, FG meagre content in SFA was significantly higher compared to SG meagre (27.79 vs. 19.21, *p* = 0.006). MUFA and ω-6 fatty acid contents were similar in both FG and SG groups. In a study on the essential fatty acid nutritional needs of juvenile meagre, Phalzgraff et al. [34] reported ω-3 (17.09%), ω-6 (13.25%), ω-3/ω-6 (1.29; ΣMUFA (43.55%), and ΣPUFA (33.73%) values in meagre flesh, which are in general agreement with those of the present study for SG and FG meagre with the exception of ΣMUFA content, which was considerably higher. Likewise, Saavedra et al. [32] reported ω-3 (15.6–19.5%), ω-6 (15.4–18.0%), ω-3/ω-6(0.9–1.1), ΣMUFA (28.2–35.0%), and ΣPUFA (33.5–38.0%) for farmed meagre of different weights.

Although fast growth and high filleting yield are primary goals in aquaculture, the present study revealed intriguing findings regarding the lipid content of slow-growing (SG) meagre. Despite the justified emphasis on rapid growth in aquaculture, the fatty acid analysis demonstrated that SG meagre exhibited a higher lipid content, which could be considered a desirable trait in terms of consumers [14]. Interestingly, the fatty acid profile of the SG fish revealed a higher content of docosahexaenoic acid (DHA), a key omega-3 polyunsaturated fatty acid known for its significant health benefits. DHA plays a vital role in supporting cardiovascular health, brain function, and development, making it a valuable nutrient for human consumption [17,19,35]. Moreover, the ratio of omega-3 (*n*-3) to omega-6 (*n*-6) fatty acids was significantly higher in SG meagre, reflecting the potential benefits of consuming this fish species. A higher n-3/n-6 ratio is often considered advantageous in terms of reducing inflammation and promoting overall health [17,19,36], further underscoring the value of SG meagre as a healthier option for human diets.

The results of the present work suggest that better growth does not always coincide with better lipid quality, challenging the common notion that faster-growing fish are always nutritionally superior. Fish metabolism and growth dynamics are crucial in shaping the fatty acid composition, as they directly influence how lipids are synthesized, stored, and utilized within the fish body [37,38,39]. Metabolic processes regulate the conversion of dietary fats into specific fatty acids, affecting the balance of ω-3 and ω-6 fatty acids, which in turn impacts the nutritional quality of the fish [40,41]. The observed fatty acid profile of both fast-growing (FG) and SG meagre indicates that SG meagre may offer enhanced nutritional value, particularly for human consumption. The higher ω-3 content along with the increased ω-3/ω-6 ratio align with dietary recommendations aimed at promoting cardiovascular health and overall well-being. Additionally, the significant increase in polyunsaturated fatty acids in SG meagre strengthens the case for its potential as a healthier alternative. Fish growth dynamics should be carefully managed in aquaculture, not only to optimize production efficiency but also to enhance the nutritional quality of the fish, particularly in terms of essential fatty acids. By understanding the interplay between growth rate, lipid accumulation, and fatty acid composition, aquaculture producers can make more informed decisions that cater to both market demands and the health needs of consumers.

### 3.3. Total Antioxidant Capacity (TOAC), SOD Activity, and Lipid Oxidation

**Figure 2:**
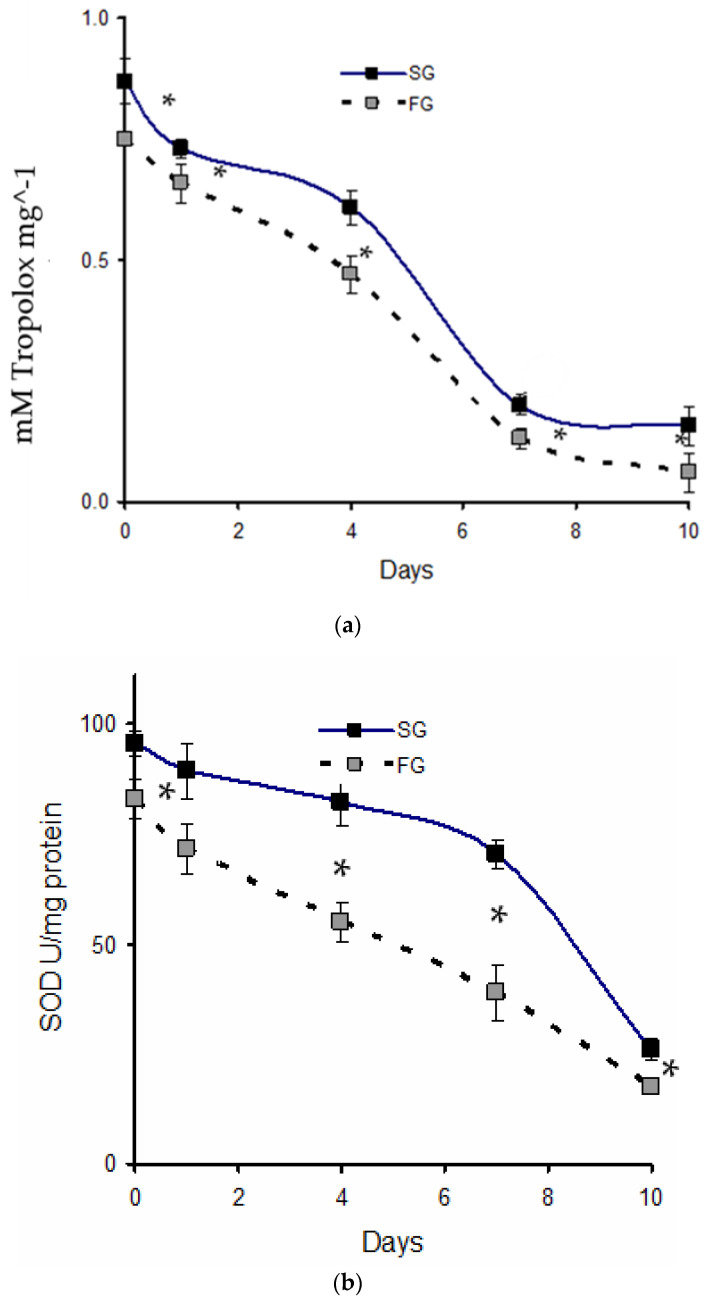

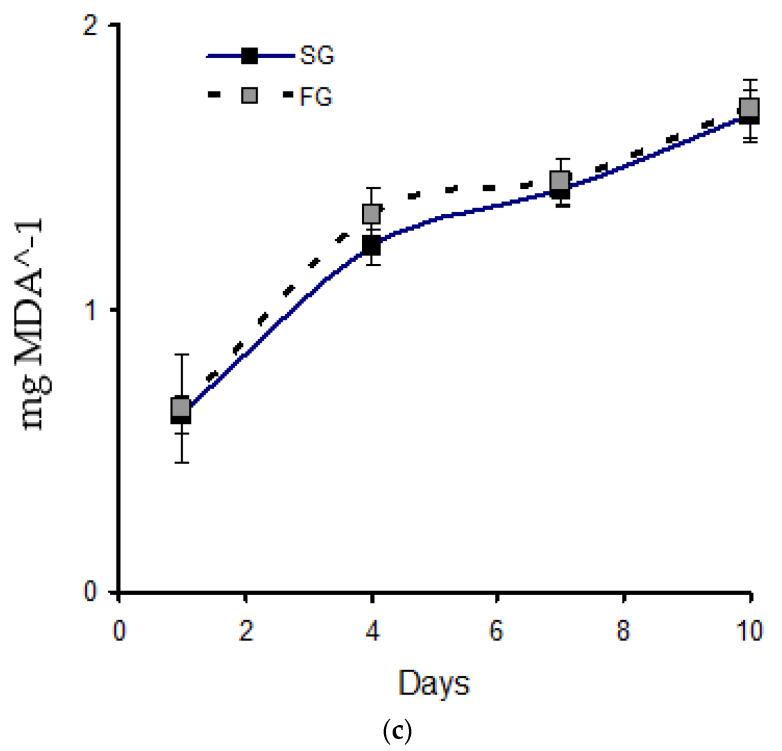
(**a**) TAOC (mM Trolox mg⁻^1^ of muscle tissue) of meagre fillets during storage at 2 °C from slow-growing (SG, grey squares, dotted line) and fast-growing (FG, black squares, solid line) meagre. Significant differences were observed between FG and SG groups on all sampling days between day 0 and day 10 (*p* < 0.05), Significant differences between FG and SG groups on specific sampling days between day 0 and day 10, as determined by Tukey’s HSD test, are marked with an asterisk (*). (**b**) SOD activity (units/mg protein) of meagre fillets during storage at 2 °C from slow-growing (SG, grey squares, dotted line) and fast-growing (FG, black squares, solid line) meagre. Significant differences were observed between FG and SG groups on all sampling days between day 0 and day 10 (*p* < 0.05), as determined by Tukey’s HSD test. (**c**) Mean (±SD) lipid oxidation values (mg MDA kg⁻^1^) of meagre fillets during storage at 2 °C from slow-growing (SG, dotted line) and fast-growing (FG, solid line) meagre. Non-significant differences were found between FG and SG groups for any sampling day (*p* > 0.05).

TAOC reflects the cumulative effect of all antioxidants, including enzymes like SOD and GPx, as well as non-enzymatic antioxidants, such as vitamins C and E. Figure 2a–c illustrate the postmortem changes in TOAC (Figure 2a), SOD activity (Figure 2b), and lipid oxidation (Figure 2c) of SG and FG meagre fillets. There was a significant effect of growth rate on TOAC, ANOVA, F(1,4) = 27.51, *p* = 0.006, *n*^2^ = 0.87. Post hoc comparisons using the Bonferroni correction revealed that the SG group exhibited significantly higher TOAC than the FG group across all sampling days (*p* < 0.05). Similarly, SOD activity was consistently higher in SG fillets compared to FG fillets across all sampling days (*p* < 0.05). Both TOAC and SOD levels decreased significantly during cold storage, with reductions of approximately 80% observed up to day 10 of storage.

In contrast to the decline in TOAC and SOD activity, malondialdehyde (MDA) levels increased in fillets from both SG and FG groups during storage at 2 °C, indicating an increase in lipid oxidation. However, unlike TOAC and SOD, which differed significantly between the two groups, no significant differences in lipid oxidation levels were observed between SG and FG fillets on any sampling day (*p* > 0.05).

The reduction in total antioxidant capacity (TOAC) observed in postmortem SG and FG meagre fish fillets during cold storage is likely due to the combined effects of antioxidant consumption in neutralizing lipid oxidation products and oxidative damage to antioxidant molecules [20,21].

MDA levels of both fillet groups remained below the threshold value of 2.0 mg MDA.kg^−1^ during the ten-day storage period, indicating minimal lipid oxidation and satisfactory freshness [42,43]. There was a gradual increase, reaching MDA values above 1.5 mg MDA kg^−1^ by day 10 (Figure 2c) yet below the limit of 2.0 mg MDA kg^−1^, signaling the onset of rancidity. This finding contrasts with the initial expectation that the higher lipid content in slow-growing (SG) fish, particularly the elevated levels of polyunsaturated fatty acids (PUFAs)—which are more susceptible to oxidation than saturated fatty acids (SFAs)—would lead to more rapid lipid oxidation. However, no significant differences in lipid oxidation were observed between SG and fast-growing (FG) fillets, which suggests that factors beyond lipid content and PUFA composition, such as intrinsic antioxidant levels, may have played a key role in mitigating oxidation. The MDA values observed in this study are comparable to those reported by Matulik et al. [13], who found oxidized tissue MDA levels in meagre ranging between 0.01 and 0.04 μmol g^−1^ (or 0.72 to 2.88 mg MDA.kg^−1^).

The differences in TAOC between SG and FG fish may be attributed to differences in their metabolism. Fast-growing fish generally exhibit higher metabolic rates to sustain rapid growth, which can increase the production of reactive oxygen species (ROS) and elevate oxidative stress [28,44,45,46,47]. Although FG fish may upregulate antioxidant production to counteract this stress, these antioxidants are likely more rapidly depleted in the process of neutralizing ROS [48,49,50]. This could at least partially explain the lower TAOC exhibited by the FG compared to SG fish, whose lower metabolic rates are expected to produce fewer ROS, probably allowing them to retain more antioxidants [49,50]. Furthermore, growth-related genes, which influence antioxidant enzyme activity, may be differently expressed in SG and FG fish, leading to varied antioxidant responses [49,51].

Meagre is known to exhibit a strong antioxidant capacity under a range of environmental and dietary regimes, particularly through the expression of key antioxidant enzymes, such as catalase, superoxide dismutase (SOD), and glutathione peroxidase (GPx) [52]. Furthermore, elevated TAOC levels are also beneficial postmortem, as antioxidant activity can slow down protein and lipid oxidation [53,54], particularly in PUFAs, which are highly susceptible to oxidative degradation.

Antioxidants can delay lipid oxidation, reducing rancidity and extending shelf life during storage. Although high TAOC and SOD values indicate a strong ability to neutralize ROS and delay lipid oxidation, their activity and, consequently, their protective effect diminished over time [20,21,54].

### 3.4. White Muscle Fiber Cellularity

**Figure 3:**
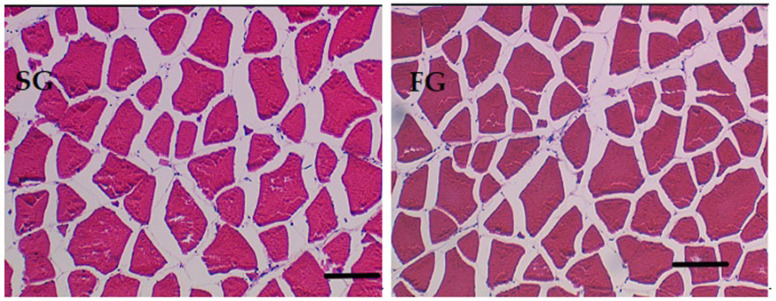
Hematoxylin and eosin (H&E) stained transverse cross-sections of white epaxial muscle tissue of slow-growing (SG) and fast-growing (FG) reared meagre displaying the arrangement and size mosaic of muscle fibers. Bar scale: 100 μm.

**Figure 4:**
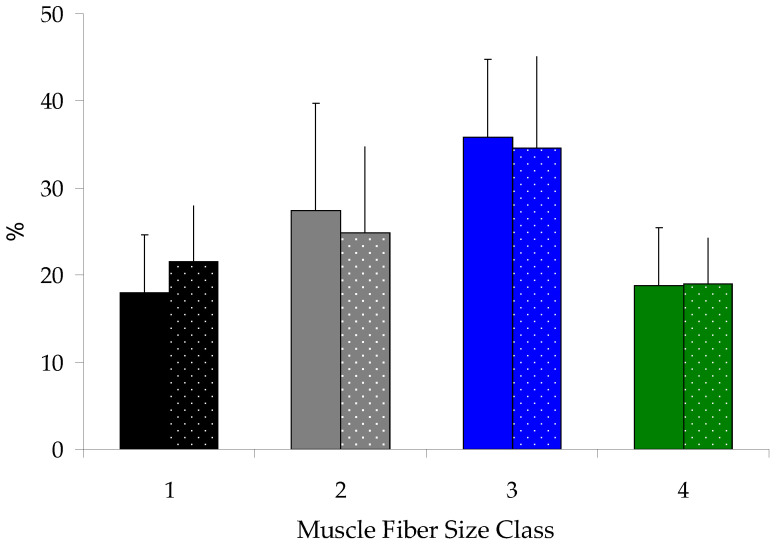
Frequency distribution (mean ± sd) of white muscle fibers cross-sectional area in the SG (solid bars) and FG (dotted bars) groups. Size classes: 1 (20–350 μm^2^); 2 (350–650 μm^2^); 3 (650–950 μm^2^); and 4 (>950 μm^2^). No significant differences were found between the two groups across any size class (ANOVA, *p* > 0.05).

Muscle cellularity was evaluated by measuring myofiber cross-sectional area (MCSA), fiber density (MFD), and size distribution of myofibers on histological sections of muscle tissue. MCSA, MFD, and size distribution values of myofibers were similar between the SG and FG groups (*t*-test (*p* > 0.05), Table 4, Figure 3 and Figure 4).

Present average fiber areas and fiber densities are in agreement with those of Saavedra [32], who reported fiber areas ranging from 0.0014 to 0.0018 mm^2^ for meagre with a range in body weight from 800 to 2500 g. Respective average fiber densities ranged from 431 to 297 fibers mm^2 −1^.

Muscle fiber characteristics may influence lipid oxidation by affecting the surface area exposed to moisture loss and oxygen within the muscle tissue, which, in turn, may influence texture and lipid oxidation during storage. Larger muscle cells (hypertrophy) can retain more moisture, resulting in less drip loss during processing and storage, which affects product textural properties [54,55]. As both groups exhibited similar muscle fiber size and density, other parameters known to affect lipid oxidation, such as differences in the fatty acid profile and intrinsic antioxidant capacity (TOAC) in SG meagre, may have contributed to the observed differences in lipid oxidation between the two groups.

Our results on muscle fiber size and density suggest that both hyperplasia and hypertrophy contributed to the observed growth differences between SG and FG meagre. This indicates that, despite differences in growth rates, SG and FG fillets shared similar muscle fiber characteristics, which may have contributed to the comparable rates of lipid oxidation despite their differing fatty acid content. Previous studies have shown that, during cold storage, muscle structure changes slowly, as observed in sea bream [15], sea bass [56], and Atlantic Herring [57].

The parameters investigated in this study—muscle cellularity, lipid profile, and oxidative stability—are crucial indices of fillet quality and are related to texture, nutritional value, freshness, and shelf life. Fillet lipid content and fatty acid profiles have been frequently reported to vary with season, size, feeding regime, and environmental conditions, all of which can, in turn, affect key parameters of fillet quality [19,20].

Our findings suggest that growth dynamics, particularly rapid growth in FG fish, may compromise the quantity and quality of fatty acids—especially EPA (eicosapentaenoic acid), DHA (docosahexaenoic acid), and other polyunsaturated fatty acids (PUFAs)—making these fillets more vulnerable to oxidative degradation during cold storage. Despite the higher lipid content in SG fish, no significant differences in MDA levels were observed between SG and FG fillets during cold storage. This may be explained by the qualitative differences in lipid profiles between the two groups combined with the antioxidant defenses shown by the higher total antioxidant capacity (TOAC) and superoxide dismutase (SOD) activity in SG fillets. The elevated TOAC in SG fillets likely played a key role in mitigating lipid oxidation, effectively counteracting the susceptibility of their PUFAs to oxidative degradation. Although TOAC and SOD levels declined over time in both groups, their higher initial levels in SG fillets may have provided greater oxidative stability, resulting in similar lipid oxidation rates between SG and FG fillets. In this context, the antioxidant capacity of fish fillets becomes a critical factor in preserving lipid stability. While SG fillets exhibited higher TOAC and SOD levels, these levels declined gradually over time in both groups, reflecting the depletion of antioxidant reserves in neutralizing reactive oxygen species. This decline was associated with reduced oxidative stability, as evidenced by increasing MDA levels during cold storage in both groups.

## 4. Conclusions

This study highlights the critical role of intrinsic antioxidants in preserving lipid quality and mitigating lipid oxidation in meagre (*Argyrosomus regius*) fillets during cold storage. The findings suggest that, despite the higher lipid content and polyunsaturated fatty acids (PUFAs) in slow-growing (SG) fish, no significant differences in lipid oxidation were observed between SG and fast-growing (FG) fish. This contrasts with initial expectations that the higher lipid content, particularly the elevated PUFAs in SG fish, would lead to more rapid lipid oxidation. Instead, the elevated total antioxidant capacity (TOAC) and superoxide dismutase (SOD) activity in SG fish likely played a key role in mitigating oxidative damage, preserving lipid stability during storage.

While no differences were found in muscle cellularity between the two groups, it is important to acknowledge the potential influence of sex-related variations on lipid metabolism, muscle structure, and post-harvest quality. Since only male fish were used in this study, future research that includes both males and females may provide further insights into the role of hormonal and metabolic differences in shaping flesh quality and antioxidant responses.

The use of 1-year-old meagre in this study is justified as fish of this age typically reach marketable sizes of 500–600 g, making them suitable for commercialization. This aligns with industry practices of size grading and allows for optimal resource utilization and revenue generation. However, further studies are needed to examine how growth dynamics, including size and age, affect not only lipid oxidation and antioxidant defense but also the broader economic and nutritional viability of meagre aquaculture.

In conclusion, this study underscores the importance of managing growth dynamics and antioxidant capacity in aquaculture to improve both the shelf life and nutritional quality of meagre fillets, especially in terms of ω-3 fatty acids. Selective breeding or dietary interventions aimed at enhancing antioxidant levels and optimizing lipid composition could contribute to improving the overall quality and sustainability of meagre farming.

## Figures and Tables

**Table 1:** Mean (±sd) values of body traits and muscle tissue proximate composition of slow-growing (SG) and fast-growing (FG) (12 months old) meagre.

Body Traits and Muscle Tissue Proximate Composition	SG	FG	*p*-Value
Body weight (BW, g)	459.09 (21.01)	1148.8 (215.32)	*
Gonadosomatic (GSI, %)	0.03 (0.04)	0.12 (0.05)	*
Hepatosomatic (HSI, %)	1.2 (0.24)	1.22 (0.25)	Nsd
Viscerosomatic (VSI, %)	4.69 (2.01)	4.78 (0.57)	Nsd
Perivisceral fat (PVF, %)	2.08 (0.25)	0.54 (0.33)	*
Filleting yield (FY, %)	31.53 (3.16)	33.46(3.53)	*
Moisture (%)	78.29 (0.78)	80.40(1.40)	*
Proteins (%)	19.0 (0.44)	18.92(0.61)	Nsd
Lipids (%)	0.78 (0.05)	0.27(0.04)	*
Ash (%)	1.10 (0.02)	1.02(0.04)	Nsd

An asterisk * indicates significant differences *p* < 0.05; Nsd, non-significant difference between FG and SG fish.

**Table 2:** Mean (±sd) fatty acid concentration (%) in muscle tissue of slow-growing (SG) and fast-growing (FG) (12 months old) meagre.

Fatty Acid	Chemical Type	SG	FG	*p*-Value
Myristic acid	C14:0	2.91 (0.75)	3.63 (1.03)	Nsd
Palmitic acid	C16:0	12.1 (1.54)	16.54 (0.92)	*
Stearic acid	C18:0	4.19 (1.26)	7.62 (0.79)	*
Palmitoleic acid	C16:1 (ω-7)	0.21 (0.19)	3.89 (0.20)	***
cis-11-octadecenoic acid	C18:1 (ω-7)	0.28 (0.25)	0.63 (0.55)	Nsd
Oleic acid	C18:1 (ω-9)	19.57 (1.48)	18.88 (2.54)	Nsd
Linoleic acid (LA)	C18:2 (ω-6)	9.83 (1.18)	8.27 (2.10)	Nsd
Linoleni cacid (LNA)	C18:3 (ω-3)	0.38 (0.11)	0.97 (0.88	Nsd
Eicosadienoic acid	C20:2 (ω-6)	0.25 (0.21)	0.82 (0.71)	Nsd
Dihomo-γ-linolenic acid (DGLH)	C20:3 (ω-6)	0.19 (0.18)	2.24 (0.46)	**
Εicosatrienoic acid	C20:3 (ω-3)	2.66 (0.52)	4.37 (0.48)	*
Arachidonic acid	C20:4 (ω-6)	3.43 (0.88)	3.71 (1.77)	Nsd
Eicosapentaenoic acid (EPA)	C20:5 (ω-3)	7.75 (8.20)	3.69 (0.45)	Nsd
Docosahexaenoic acid (DHA)	C22:6 (ω-3)	13.90 (3.31)	7.47 (1.11)	*
Nervonic acid	C24:1 (ω-9)	7.81 (0.79)	7.25 (1.24)	Nsd

An asterisk * indicates significant differences between groups: * *p* < 0.05; ** *p* < 0.01; *** *p* < 0.001. Nsd, non-significant differences.

**Table 3:** Mean (±sd) concentration (%) of lipid quality indices in white muscle of slow-growing (SG) and fast-growing (FG) (12 months old) meagre.

Lipid Quality Indices	SG	FG	*p*-Value
ω-3	24.30 (1.45)	15.52 (1.26)	**
ω-6	13.70 (1.39)	15.04 (3.00)	nsd
ω-3/ω-6	1.78 (0.14)	1.07 (0.30)	*
SFA	19.21 (2.66)	27.79 (0.95)	**
ΣMUFA	27.88 (1.72)	29.86 (1.37)	nsd
ΣPUFA	38.38 (31.53)	31.53 (1.22)	*

SFA: saturated fatty acids, MUFA: monounsaturated fatty acids, PUFA: polyunsaturated fatty acids. Asterisk indicates significant differences: * *p* < 0.05, ** *p* < 0.01. nsd: non-significant differences.

**Table 4:** Mean (±sd) values of myofiber cross-sectional area (MCSA) and density (MFd) in meagre white muscle.

Myofiber Characteristics	SG	FG	*p*-Value
MCSA (μm^2^)	1516.42 (±63.7) [0.0015 mm^2^]	1667.44 (±448.9) [0.0017 mm^2^] [0.0017 mm^2^]	nsd
MFd (number/mm^2^)	510.48(±131.8)	449.68 (±106.8)	nsd

Numbers in brackets indicate the MCSA expressed in mm^2^. nsd: non-significant differences.

## Data Availability

Dataset available on request from the authors.
